# Proapoptotic Activity of a Monomeric Smac Mimetic on Human Fibroblast-Like Synoviocytes from Patients with Rheumatoid Arthritis

**DOI:** 10.1007/s10753-014-0012-1

**Published:** 2014-09-12

**Authors:** D. Lattuada, C. Casnici, K. Crotta, P.F. Seneci, C. Corradini, M. Truzzi, F. Ingegnoli, O. Marelli

**Affiliations:** 1 Department of Medical Biotechnology and Translational Medicine, University of Study, Milan, Italy; 2 Department of Chemistry, University of Study, Milan, Italy; 3 Department of Biomedical Sciences, Surgical and Dental, School of Medicine, University of Study, Milan, Italy; 4 Division of Rheumatology, Istituto Gaetano Pini, Department of Clinical Sciences & Community Health, University of Study, Milan, Italy

**Keywords:** rheumatoid arthritis, Smac mimetics, fibroblast-like synoviocytes, IAP proteins, synovium, osteoarthritis

## Abstract

Inhibitors of apoptosis proteins (IAPs) block cell death in response to diverse stimuli. The mitochondrial protein, second mitochondria-derived activator of caspase (Smac), negatively regulates IAP inhibition of caspase activity. We investigated the proapoptotic activity of a synthetic Smac (Smac 066) on fibroblast-like synoviocytes (FLS) derived from patients with active rheumatoid arthritis (RA). We found that Smac 066 induced significant apoptosis in all RA-FLS samples. Furthermore, IAPs, which are upregulated in RA-FLS, were downregulated by Smac 066. This suggested that IAPs upregulation was responsible for RA-FLS sensitivity to Smac 066. Next, we analysed caspase activation and found that Smac 066 was associated with caspase 8 and caspase 3 activities. We then investigated the mechanism underlying Smac 066 downregulation of IAPs in RA-FLS with an apoptotic pathway array. Interestingly, Smac 066 significantly upregulated IGFBP-5, a protein involved in differentiation, apoptosis, and osteoblastic activation. Smac 066 may represent a new therapeutic approach to RA treatment.

## INTRODUCTION

The inhibitors of apoptosis proteins (IAPs) comprise a family of proteins involved in the regulation of various cellular processes, including apoptosis. IAPs may also contribute to tumour growth because their expression is increased in tumour cells. These proteins are characterised by the presence of one to three baculoviral IAP repeat (BIR) domains [1,2]. BIR domains are primarily responsible for the anti-apoptotic activity of IAPs because they bind to caspases 3, 7, and 9 and prevent assembly of caspases 8 and 10. The anti-apoptotic activity of IAP proteins can be blocked by the second mitochondria-derived activator of caspases (Smac), which is liberated into the cytoplasm in response to proapoptotic stimuli [3,4]. Several monomeric and dimeric compounds have been synthesised to imitate the structure of these molecules (Smac mimetics). The mimetic structures were designed to resemble the N-terminal amino acid residues (AVPI) of the mitochondrial Smac protein [4]. These compounds were shown to bind specifically to three members of the IAP family: XIAP, cellular IAP1 (cIAP1), and cellular IAP2 (cIAP2). These mimetic molecules are currently under investigation as potential anti-neoplastic drugs, and they have shown promise in clinical trials as an anti-cancer therapy [5,6].

In addition, several inflammatory diseases, such as rheumatoid arthritis (RA), are characterised by hyperplastic invasive tissue, which resembles tumour growth; this invasion causes progressive destruction of the diarthrodial joints and leads to severe disability [7, 8]. In RA, the synovium transforms from a relatively quiescent cellular structure to a hyperplastic, invasive tissue, called ‘pannus’. Pannus forms at the cartilage–bone interface, where it cloaks the cartilage and erodes the bone [9]. Pannus is composed of macrophages, osteoclasts, and invasive synovial lining fibroblasts (or fibroblast-like synoviocytes [FLS]), with relatively few lymphocytes. RA-FLS contribute to the local production of cytokines and proteolytic enzymes, which degrade the extracellular matrix. The RA-FLS have an unusually aggressive, tumour-like behaviour phenotype that results from exposure to the toxic rheumatoid synovial environment. Furthermore, several aspects of RA-FLS that resemble those of tumours have been described, including somatic mutations in proto-oncogenes, increased expression of anti-apoptotic molecules, decreased response to receptors that activate apoptosis, and abnormalities in the p53 pathway [10]. A few attempts have been made to modulate RA-FLS activity; among these, the induction of apoptosis appears to be the most promising [11, 12].

Because Smac mimetics have been shown to be effective on cancer cells, and RA-FLS have many properties similar to cancer cells [13], we reasoned that Smac mimetics may also be effective in RA-FLS cells, which are resistant to apoptosis. Proper regulation of apoptosis may be crucial for the development and maintenance of health in patients with RA [14]. Thus, our specific objective was to examine whether the Smac mimetic compound, Smac 066, showed proapoptotic activity in RA-FLS.

## METHODS

### Patients and Tissue Collection

Synovial tissue was obtained from patients with RA (*n* = 10) or osteoarthrosis (OA; *n* = 5) during joint synovectomies. All patients provided written informed consent. The use of these tissues for research was approved by the hospital ethics committee. Synovial tissues were cut into small pieces and digested with collagenase (Sigma-Aldrich, St Louis, MO) in Dulbecco’s modified Eagle’s medium (DMEM) for 2 h at 37 °C to isolate FLS. The FLS were grown in DMEM containing 10 % FetalClone 1 (Thermo Scientific, USA), 100 U/ml penicillin, and 100 mg/ml streptomycin (Sigma-Aldrich, St Louis, MO) in a humidified incubator at 37 °C under 5 % CO_2_. At confluence, the cells were trypsinised and passaged; all cells were used in experiments between passages 3 and 8.

### Smac Mimetic Compounds

**Fig. 1:**
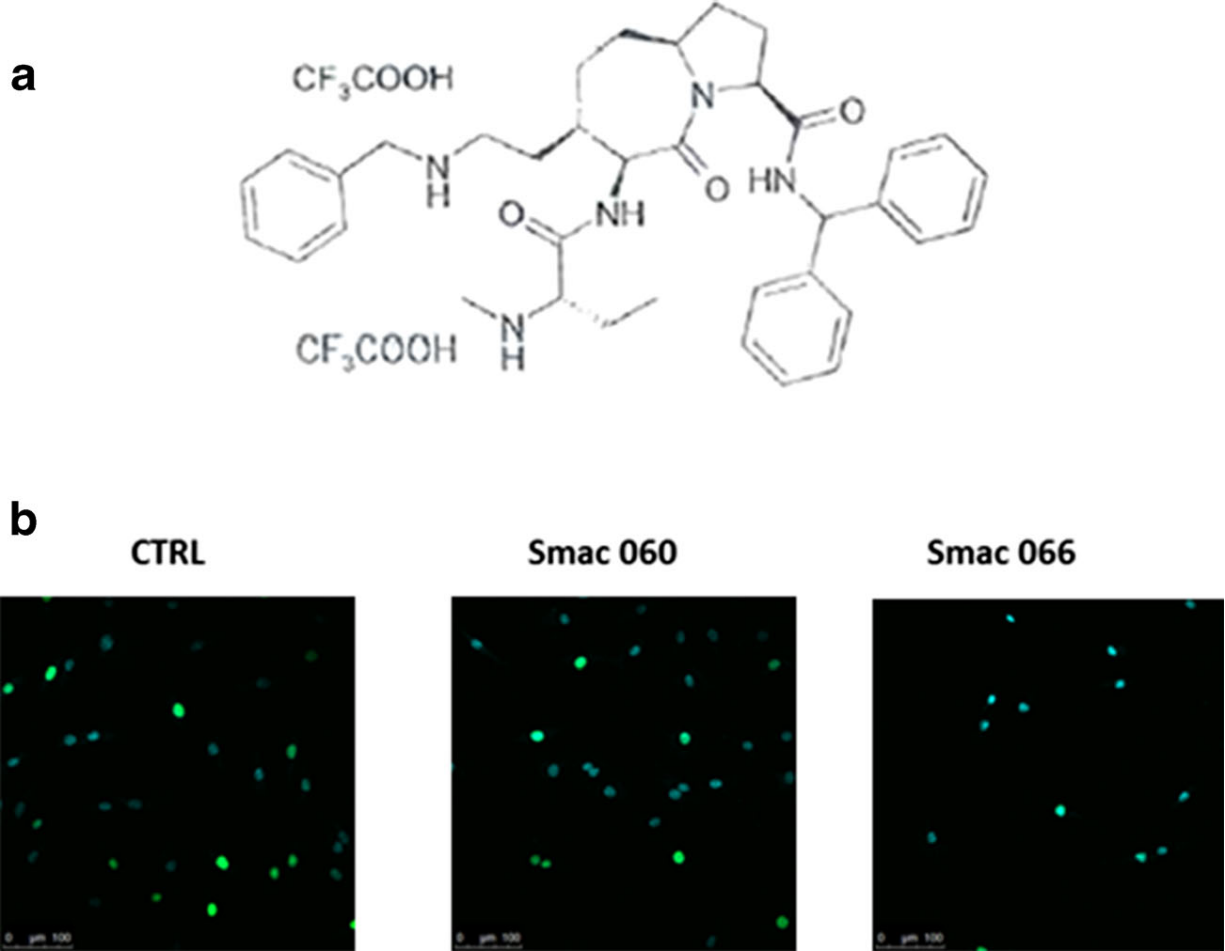
Smac 066 promotes apoptosis in fibroblast-like synoviocytes (FLS) derived from patients with rheumatoid arthritis (RA). **a** Structural formula of Smac 066. **b** RA-FLS were cultured in the presence of Smac 060 or 066 (20 M, overnight) or tissue medium alone (*CTRL*). DNA synthesis was allowed to proceed in a 24-h incubation with 10 μM 5-ethynyl-2′-deoxyuridine (EdU conjugated to Alexa Fluor 488). Cells were then fixed and permeabilised, and EdU incorporated into newly synthesised DNA was detected with fluorescence confocal microscopy. Confocal microscopy images of a representative experiment are shown: *green*—newly synthesised DNA, *blue*—Hoechst staining of total DNA.

We tested ten different monomeric Smac mimetic compounds (unpublished data), and we chose to include 060 and 066 (Fig. 1) in the current study. The synthesis of Smac was carried out as described elsewhere [15, 16]. Smac molecules were dissolved and diluted in water. Smac 060 is ineffective on fibroblasts; thus, it was used as a negative control (US Patent Application No: 2008/0021,066).

### Proliferation Assay

FLS were cultured in the presence of Smac 060 or 066 (20 μM, overnight) or in tissue medium alone (CTRL). DNA synthesis was evaluated by adding 10μ M 5-ethynyl-2-deoxyuridine (EdU) labelled with Alexa Fluor 488 dye (Click-iT® EdU Alexa Fluor® 488 HCS Assay, Invitrogen, Carlsbad, CA). After 24-h incubations, cells were fixed and permeabilised. We evaluated EdU incorporated into newly synthesised DNA by detecting the fluorescent Alexa Fluor 488 label. Analysis was performed with fluorescence confocal microscopy.

### Apoptosis Assay

RA-FLS were treated with 20μM Smac 060 or 066 for 18 h. Apoptotic cells were detected with the Annexin V-FITC apoptosis detection kit (Abcam, Cambridge, UK), according to the manufacturer’s instructions. All samples were analysed with a FACSCalibur flow cytometer equipped with an argon laser at 488 nm (Becton Dickinson). FITC-conjugated Annexin V emission was collected in the FLH-1 channel, and propidium iodide (PI; for detecting necrotic or dead cells) emission was collected in the FLH-3 channel. Data were analysed with CellQuest software. The percentage of apoptosis was calculated, considering cells in both early (Annexin^+^ PI^−^) and late apoptosis (Annexin^+^ PI^+^).

### Western Blots

RA- and OA-FLS were treated with 20 μM Smac 060 or 066 for 18 h. Whole cell extracts were then prepared by directly lysing the cells in lysis buffer. Cell lysates were processed and the protein concentration was measured with the BCA method (Thermo Scientific, USA), according to the manufacturer’s instructions. Proteins in the cell lysates were separated with SDS–PAGE on 4–12 % Tris–HCl precast gels (Invitrogen, Carlsbad, CA). Proteins in the gel were transferred onto nitrocellulose membranes (Invitrogen, Carlsbad, CA). The membranes were blocked for 3 h with 5 % non-fat dry milk (Lab Scientific) in 0.1 % PBS–Tween 20. Then, membranes were incubated with a primary antibody overnight at 4 °C. Next, we added secondary antibodies conjugated to horseradish peroxidase (Thermo Scientific, USA), and membranes were developed with Western Lightning Plus ECL (PerkinElmer, OH, USA). Densitometry was performed with ImageJ software (National Institutes of Health, Bethesda, USA). We used primary antibodies to the following proteins: cIAP1 (R&D Systems, Minneapolis), cIAP2 (BD Pharmingen, MA, USA), and XIAP (Cell Signalling Technology).

### Apoptosis Array

Apoptosis was induced by adding 20 μM Smac 066 to cultures for 18 h. Then, 2 × 10^7^ RA-FLS were solubilised in lysis buffer containing a protease inhibitor cocktail. The proteins concentration of the samples was determined with the BCA method (Thermo Scientific, USA). We evaluated the presence of 96 apoptotic proteins with an Apoptosis Array Kit (Ray Biotech), according to the manufacturer’s instructions.

### Caspase Detection

RA-FLS and OA-FLS were cultured in DMEM medium containing 10 % FetalClone 1 serum (Thermo Scientific, USA), 100 U/ml penicillin, and 100 mg/ml streptomycin (Sigma-Aldrich, St Louis, MO) in a humidified incubator at 37 °C under 5 % CO_2_. Apoptosis was induced in the cells by adding 15 μM staurosporine (Sigma-Aldrich, St Louis, MO) or 20 μM Smac 060 or Smac 066 for 6 h. FLS were then collected for Western blot analysis. To detect caspases, we used an anti-caspase 3 rabbit monoclonal antibody (Cell Signalling Technology) and an anti-caspase 8 antibody (Cell Signalling Technology); β-actin (Sigma-Aldrich, St Louis, MO) was used as the loading control.

### Statistical Analysis

All data are expressed as the mean ± standard error of the mean. Statistical analyses were carried out with one-way analysis of variance and Student’s *t* tests.

## RESULTS

### Smac 066 in RA-FLS

Seneci *et al.* designed Smac 066 to enhance lipophilicity and promote intracellular uptake. Therefore, this compound possesses a methyl group attached to the terminal amine and the 4-substituent in the central ring was elongated to produce a more apolar arm (Fig. 1a) [15]. To study the effect of Smac 066, we cultured RA-FLS in the presence of this compound, and we evaluated proliferation (Fig. 1b). We found that RA-FLS growth was significantly inhibited in the presence of Smac 066 compared to cells grown in tissue medium alone (CTRL) or in the presence of Smac 060, the negative control. Smac 060 is a monomeric compound structurally similar to Smac 066, but it has no proapoptotic activity (data not shown). Smac 060 did not substantially affect apoptosis because it lacks a 4-substitution on the 1-2aza-oxobicyclo [5.3.0] decane scaffold; this 4-substitution, which is present in Smac 066, establishes a novel molecular interaction with binding sites on XIAP, and it contributes to 066 solubility and penetration through biological membranes.

### Smac 066 Induces Apoptosis in RA-FLS

**Fig. 2:**
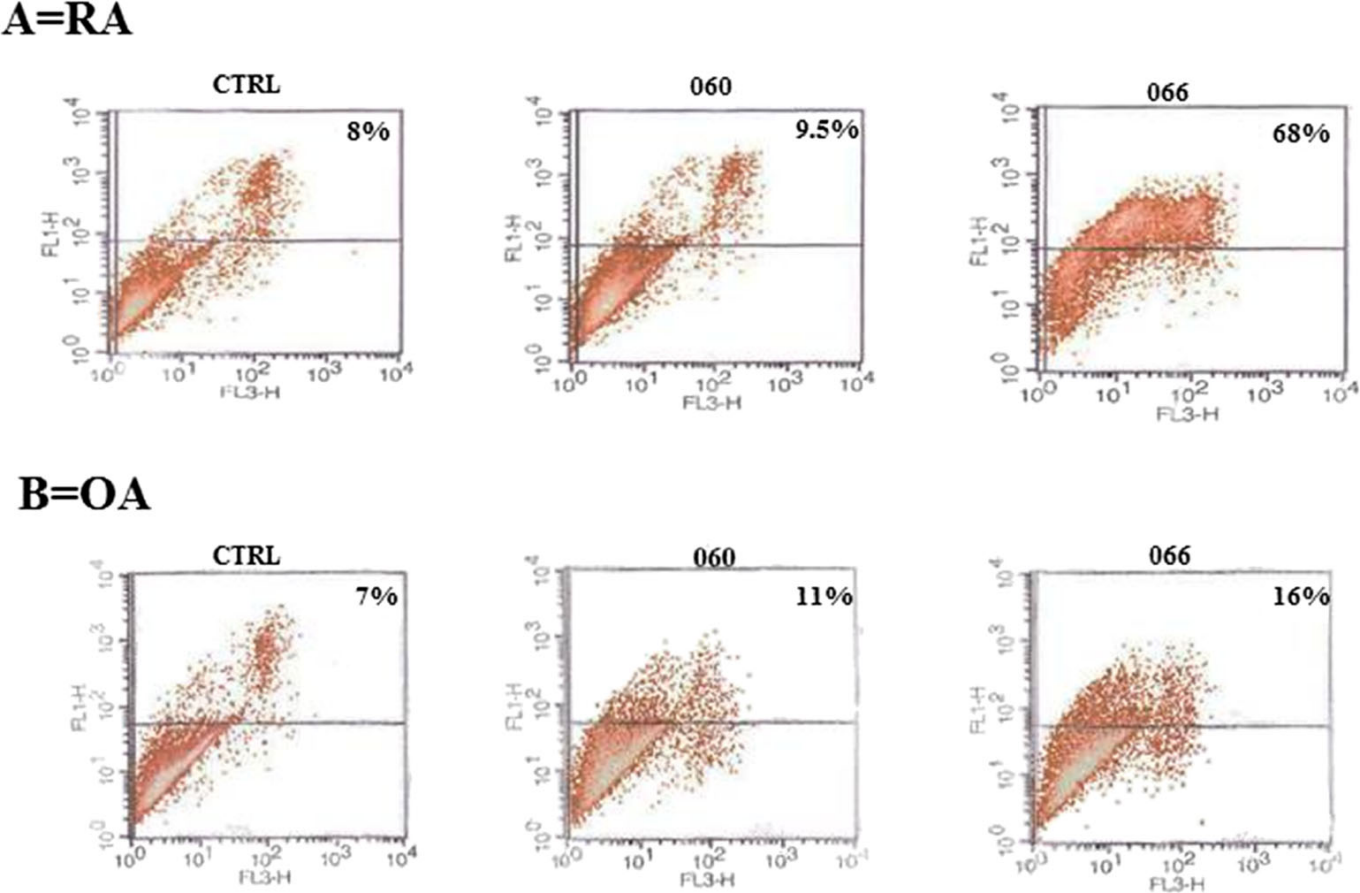
Flow cytometry results show apoptosis, determined with the Annexin V test. **a** Fibroblast-like synoviocytes (FLS) derived from patients with rheumatoid arthritis (*RA*) were incubated overnight with 20 μM Smac 060 or 066 or tissue medium alone (*CTRL*). **b** FLS from patients with osteoarthritis (*OA*) were incubated overnight with 20 μM Smac 060 or 066 or tissue medium alone (*CTRL*). *Ordinal axes*, Annexin^+^ cells; *abscissa axes*, propidium iodide (PI)^+^ cells. Data are expressed as percentage of Annexin^+^ + Annexin^+^ PI^+^ cells. Data are representative of four independent experiments.

We investigated whether Smac 066 could induce apoptosis in RA-FLS compared to OA-FLS. We isolated FLS from the synovial tissues of patients with RA or OA and cultured them in the presence of either Smac 066 or Smac 060. As shown in Fig. 2a and b, both Annexin V^+^ PI^−^ FLS (*i.e.*, early apoptotic) and Annexin V^+^ PI^+^ FLS (*i.e.*, late apoptotic) signals were detected in RA- and OA-FLS. The results clearly demonstrated that only RA-FLS treated with Smac 066 underwent significant apoptosis; in contrast, the cellular status of OA-FLS did not change (Fig. 2b). Moreover, Smac 060 was not effective on either RA-FLS or OA-FLS (Fig. 2a, b).

### IAPs Downregulated by Smac 066 in RA-FLS

**Fig. 3:**
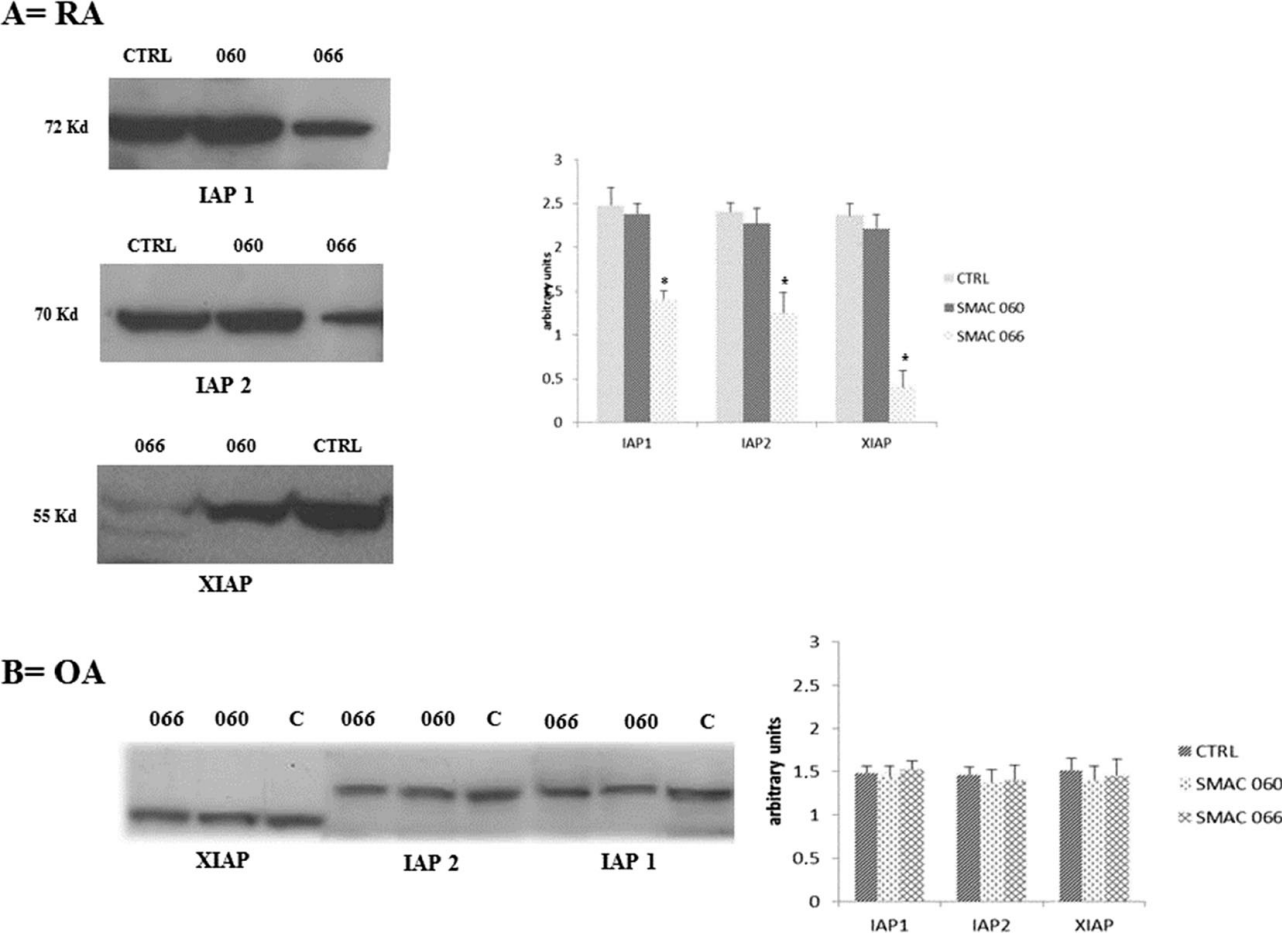
Western blots show Smac 060 and 066 effects on IAPs levels in fibroblast-like synoviocytes (FLS) derived from patients with rheumatoid arthritis (RA-FLS) or osteoarthritis (OA-FLS). *Left panels*—immunoblots show detection of cIAP1 (72 kDa), cIAP2 (70 kDa), and XIAP (55 kDa). Actin was used as a loading control (42 kDa; not shown). *Right panels*—densitometric analyses of the immunoblots show the ratio of IAPs/actin protein expression ± standardised error of the mean from four independent experiments. *P < 0.05 indicates statistically significant differences compared to untreated RA-FLS (CTRL or C).

Smac mimetics are potent IAP antagonists; therefore, we evaluated the expression levels of IAPs after 18 h of treatment with Smac 066 and 060. As shown in Fig. 3a, Western blotting and densitometric analysis showed that only Smac 066 significantly downregulated cIAP1, cIAP2, and XIAP in RA-FLS. In contrast, the levels of these proteins in OA-FLS extracts did not change after treatment with the same Smac compounds. The lack of Smac 066 action on OA fibroblasts probably resulted because IAPs are not upregulated in OA-FLS (Fig. 3b).

### IGFBP-5 Upregulated by Smac 066 in RA-FLS

**Fig. 4:**
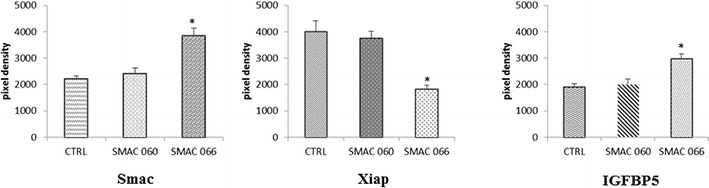
Human apoptotic protein-array results show differences in the release of the indicated proteins from untreated and treated fibroblast-like synoviocytes (FLS) derived from patients with rheumatoid arthritis (RA). Autoradiographs of the arrays were scanned to determine the density of the protein-array positions. Values from scans were adjusted based on the intensity of control spots on the filter corners; the increases in densities of specific proteins are shown. This is one representative series of arrays from two experimental repeats.

Based on the finding that Smac 066 had an effect on RA-FLS, we sought to determine whether Smac 066 could induce the expression of other proteins involved in the apoptotic pathway. With an apoptosis array kit, 96 proteins in the apoptotic pathway were analysed by Western blot. In RA-FLS treated with Smac 066, we observed downregulation of XIAP and upregulation of endogenous Smac and insulin-like growth factor binding protein 5 (IGFBP-5) (Fig. 4). The latter protein is the most conserved in the IGFBP family. IGFBP-5 has several regulatory functions, and it is involved in various cell phenomena, including proliferation, differentiation, apoptosis, and osteoblastic activation [17].

### Cleavage of Caspases in RA-FLS by Smac 066

**Fig. 5:**
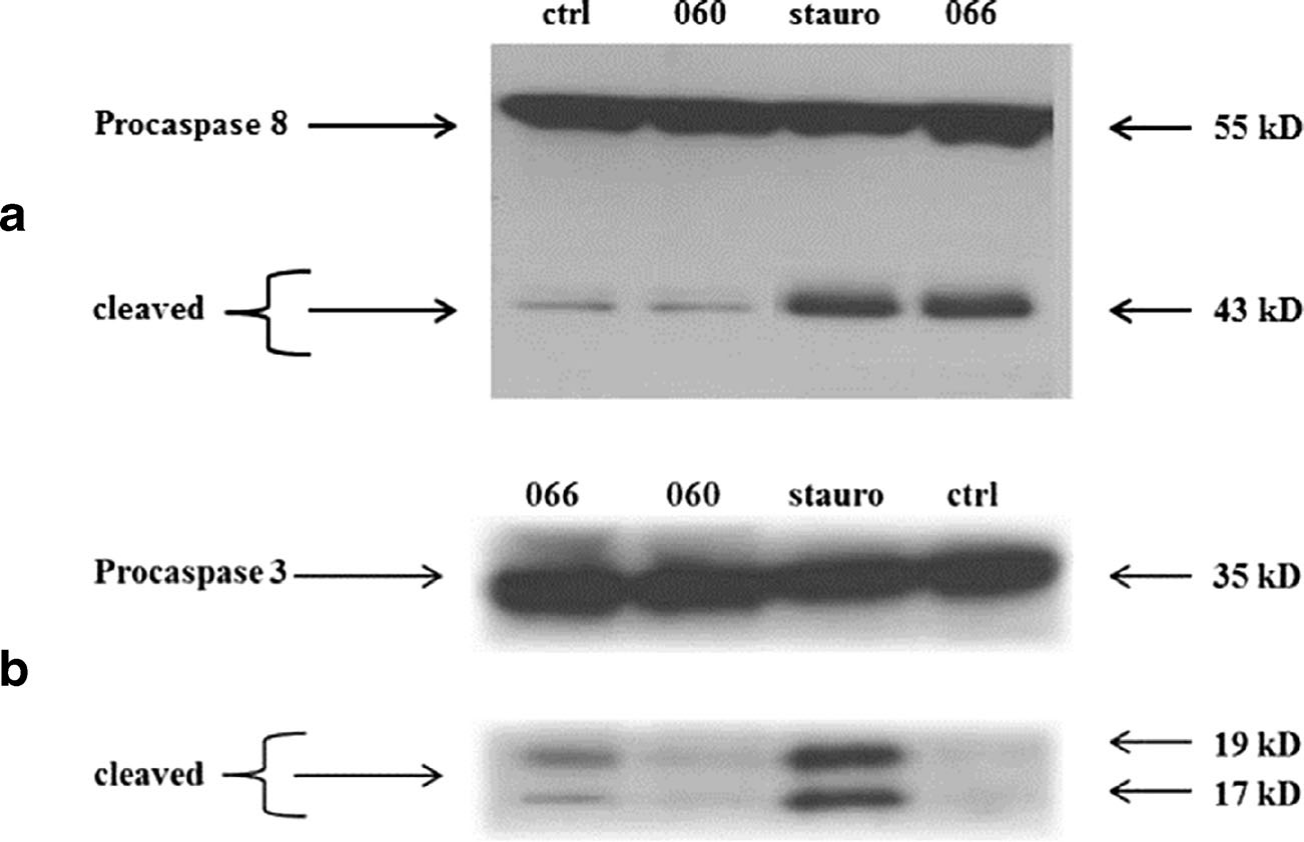
Western blots show the effect of Smac 066 on caspases in extracts from human fibroblast-like synoviocytes (FLS) derived from patients with rheumatoid arthritis (RA). Levels of cleaved caspase 8 (**a**) and cleaved caspase 3 (**b**) were measured in RA-FLS untreated (*ctrl*) or treated with Smac 060, Smac 066, or with the apoptosis inducer staurosporine (*stauro*). Cells were incubated for 6 h with 15 μM staurosporine as a positive control.

To confirm our results regarding Smac 066 activity, we analysed the activation of caspases 3 and 8 after incubating RA-FLS with Smac 066 for 6 h. As shown in Fig. 5, both caspases were cleaved. As a positive control for this assay, RA-FLS were incubated with the apoptosis inducer, staurosporine, for 6 h.

## DISCUSSION

RA is a chronic disease that causes pain, swelling, and stiffness; it limits the range of motion and reduces function in the affected joints. Although the joints are the most involved parts of the body, inflammation can also develop in the internal organs (such as lungs, kidneys, heart, nervous system, eyes). Unfortunately, many patients with RA are resistant to conventional therapies; therefore, it is important to find new molecules that, alone or in association with classic drugs, will be effective in treating this pathology.

RA-FLS play pivotal roles in both the initiation and perpetuation of RA. These cells have been prominently linked to the progressive destruction of articular structures, particularly cartilage [18, 19]; moreover, they are the principal cells responsible for RA hyperplasia because they are resistant to apoptosis. This resistance depends on IAPs upregulation [20]. The anti-apoptotic activity of IAPs can be blocked by the mitochondrial protein, Smac, which is liberated into the cytoplasm in response to proapoptotic stimuli [3,4]. Several Smac-mimetic compounds have been synthesised in drug development. Previous studies used computational, NMR, and X-ray data to establish a preliminary structure–activity relationship for XIAP inhibitors, and this led to the design of optimised analogues [15,16]. Smac mimetics can target different members of the IAP family and cause proteasomal-dependent degradation of cIAP1 and cIAP2 [13]. These Smac compounds are currently being investigated as potential anti-neoplastic drugs, and they have shown promise in clinical trials as an anti-cancer therapy [5,6]. However, these compounds have not been tested in autoimmune diseases.

In this study, we used Smac 066 (synthesised by Seneci *et al.*) because, compared to earlier compounds, it was enhanced to improve membrane permeation [15]. An important goal of our study was to demonstrate that Smac 066 could effectively induce apoptosis in RA-FLS. The Annexin V test was used to probe apoptosis because an important hallmark of apoptosis is the exposure of phosphatidylserine residues on the outer leaflet of the plasma membrane. Our experiments demonstrated that Smac 066 could induce apoptosis in RA-FLS, but not in OA-FLS. One explanation could be that, although apoptosis resistance is mediated by IAPs upregulation in RA-FLS, the apoptosis resistance in OA-FLS is independent of IAPs upregulation. In OA, apoptosis resistance is correlated to exposure to pro-inflammatory cytokines, IL-1 and TNF-α, which disturb normal mitochondrial function in human OA cells by causing mitochondrial DNA damage. This damage leads to reduced energy production and reduced mitochondrial transcription [21,22]. The downregulation of IAPs by Smac 066 was confirmed in our Western blot analysis, where we observed decreased levels of cIAP1, cIAP2, and XIAP only in RA-FLS extracts treated with monomeric Smac 066.

Then, because IAPs are involved in complex cell signalling pathways [23], we analysed a group of essential apoptotic molecules to improve our understanding of the mechanism of action of Smac compounds in RA-FLS. We observed an increase in endogenous Smac and IGFBP-5 in RA-FLS treated with Smac 066. The function of IGFBP-5 in the RA synovium is not fully understood, but it seems to be involved in the promotion of osteoblastic activity and bone formation [24,25]. Thus, in addition to the proapoptotic action of Smac 066, the upregulation of IGFBP5 could reduce osteoclast activity, which is highly important in the pathogenesis of RA.

We next studied Smac activity on caspase activation in RA-FLS. We demonstrated that both caspase 3 and caspase 8 were cleaved. Thus, Smac could promote both the proteolytic activation of pro-caspase 3 and the enzymatic activity of mature caspase 3. These activities depended on the ability of Smac to specifically interact with XIAP. In fact, Smac 066 interfered with the XIAP binding site of caspase 3; thus, it promoted both extrinsic and intrinsic programmed cell death [3]. On the other hand, the inhibition of IAPs could activate TNF-α pathways. Indeed, several members of the TNF family of cytokine receptors recruit caspase 8 to their cytosolic domains; this recruitment results in the proteolytic activation of the caspase pathway [26]. Considering our results and those of previous studies, it is possible that caspase 3 activation is induced by a direct interaction between Smac and XIAP molecules; however, Smac activation of caspase 8 implies an involvement of the TNF-α receptor 1 signalling complex [27, 28].

In conclusion, we demonstrated that Smac 066 could effectively induce apoptosis in RA-FLS, but not in OA-FLS. This proapoptotic activity may be attributed to the inhibitory effects of Smac 066 on IAPs and to its ability to activate caspase pathways. Thus, our findings suggested that Smac 066 is a promising, potential local therapeutic approach to RA. Local therapeutic approaches, combined with systemic immunosuppressive agents, may facilitate more effective control of disease activity and progression.

## Non-standard Abbreviations

AVPI: N-terminal amino acid residues
BIR: Baculoviral IAP repeats
EdU: 5-Ethynyl-2-deoxyuridine
FLS: Fibroblast-like synoviocytes
IAPs: Inhibitor of apoptosis proteins
OA: Osteoarthritis
OA-FLS: Osteoarthritis fibroblast-like synovial cells
RA: Rheumatoid arthritis
RA-FLS: Rheumatoid arthritis fibroblast-like synovial cells
RIP2: Receptor-interacting protein 2
Smac: Second mitochondria-derived activator of caspase
